# Exploring the Privacy-Preserving Properties of Word Embeddings: Algorithmic Validation Study

**DOI:** 10.2196/18055

**Published:** 2020-07-15

**Authors:** Mohamed Abdalla, Moustafa Abdalla, Graeme Hirst, Frank Rudzicz

**Affiliations:** 1 Department of Computer Science University of Toronto Toronto, ON Canada; 2 The Vector Institute for Artificial Intelligence Toronto, ON Canada; 3 Institute for Clinical Evaluative Sciences Toronto, ON Canada; 4 Deptartment of Statistics Computational Statistics & Machine Learning Group University of Oxford Oxford United Kingdom; 5 Wellcome Centre for Human Genetics Nuffield Dept of Medicine University of Oxford Oxford United Kingdom; 6 Harvard Medical School Boston, MA United States; 7 International Centre for Surgical Safety Li Ka Shing Knowledge Institute St Michael’s Hospital Toronto, ON Canada; 8 Surgical Safety Technologies Inc Toronto, ON Canada

**Keywords:** privacy, data anonymization, natural language processing, personal health records

## Abstract

**Background:**

Word embeddings are dense numeric vectors used to represent language in neural networks. Until recently, there had been no publicly released embeddings trained on clinical data. Our work is the first to study the privacy implications of releasing these models.

**Objective:**

This paper aims to demonstrate that traditional word embeddings created on clinical corpora that have been deidentified by removing personal health information (PHI) can nonetheless be exploited to reveal sensitive patient information.

**Methods:**

We used embeddings created from 400,000 doctor-written consultation notes and experimented with 3 common word embedding methods to explore the privacy-preserving properties of each.

**Results:**

We found that if publicly released embeddings are trained from a corpus anonymized by PHI removal, it is possible to reconstruct up to 68.5% (n=411/600) of the full names that remain in the deidentified corpus and associated sensitive information to specific patients in the corpus from which the embeddings were created. We also found that the distance between the word vector representation of a patient’s name and a diagnostic billing code is informative and differs significantly from the distance between the name and a code not billed for that patient.

**Conclusions:**

Special care must be taken when sharing word embeddings created from clinical texts, as current approaches may compromise patient privacy. If PHI removal is used for anonymization before traditional word embeddings are trained, it is possible to attribute sensitive information to patients who have not been fully deidentified by the (necessarily imperfect) removal algorithms. A promising alternative (ie, anonymization by PHI replacement) may avoid these flaws. Our results are timely and critical, as an increasing number of researchers are pushing for publicly available health data.

## Introduction

### Motivation

Natural language processing (NLP) is increasingly used to assist medical practitioners with various tasks, ranging from patient phenotyping to unplanned hospital readmission prediction [1-3]. Although a diverse range of approaches are used, a large number of NLP applications use algorithms, such as Continuous Bag of Words (CBOW), Skipgram, and Global Vectors (GloVe) [4,5], which represent tokens as dense numeric vectors termed as *word embeddings.* Most of these representations are computed from large corpora of text, such as clinical notes or narratives from health records, made available by health care providers (HCPs). Usually, before these data are provided to researchers, the HCPs apply anonymization algorithms to deidentify the personal health information (PHI) in the data. In this work, we adopted the terminology of the US Health Insurance Portability and Accountability Act (HIPAA), where *PHI* refers to *individually identifiable health information*, which includes personal identifiers ranging from names and phone numbers to fingerprints.

There is a wide variety of techniques to locate and deidentify PHI in clinical text, ranging from dictionaries [6] to recurrent neural networks [7]. Once the sensitive information is located within a record, anonymization can employ either *removal* or *replacement*, that is, the sensitive information is either simply deleted, changed to a data-type identification tag such as *NAME*, or replaced with another randomly chosen PHI of the same type. Many publicly available resources use PHI removal, for example, the Multiparameter Intelligence Monitoring in Intensive Care (MIMIC-III) dataset [8] used informative deidentification tags. However, in this paper, we showed that as no perfect PHI search algorithm exists, data *secured* this way can be exploited because traces of identities remain in the text and are detectable even in embeddings that are generated from it.

Specifically, we discussed the privacy concerns that arise from publicly releasing word embeddings that have been trained on clinical notes secured using the PHI removal paradigm. At first glance, it may seem that releasing word embeddings has low risk because of the unordered nature of these models; all that is released is a list of words, arbitrarily ordered, with dense numeric vectors associated with each word. However, through our experiments with three of the most popular embedding techniques, we showed that they can be leveraged to learn information presumed to be removed.

Our work relies on the assumption that some name tokens will inevitably be missed by the deidentification process. This is a realistic assumption as, to date, there is no deidentification algorithm that has perfect recall (ie, captures all PHI). This necessarily means that the word list of the embedding model will contain names that are not properly protected. We also assume that malicious actors will be able to successfully identify these tokens from a very large wordlist. Given these two assumptions, a publicly released traditional word embedding model then presents a small, but nontrivial, risk of patient identities being attacked. This risk is relative to the number of patients in the data set and the particular deidentification and embedding algorithms used. Up to 0.6% of all patients may be at risk of having their full names detected in a data set (built from individual name tokens), and as many as 0.02% to 0.15% may have their full name associated with a diagnosis. Although these risks appear small, with the growing number of publicly available embeddings trained on clinical data, we aimed to draw attention to the possible critical mass of potential privacy exposure.

Specifically, we showed that (1) it is possible to associate name tokens together to form *true* name pairs, (2) there is a significant difference between the distances of diagnoses that have been associated with a patient and those of diagnoses not associated, and this is true both at the population level and at the patient level, and (3) it is possible for a malicious actor to determine diagnoses assigned to multiple patients, using only precomputed embeddings. In this work, we will refer to diagnostic codes and diagnoses interchangeably, although this is not, of course, a general equivalence. Here, we take the diagnostic code simply as an indication of the condition that the patient is suspected of having, which is sensitive information that must be protected. Finally, we replicate these results and perform further experiments with a synthetic data set that we make publicly available.

Our work is the first to study the privacy implications of releasing word embeddings. This demonstrates how anonymizing clinical notes using PHI removal is likely to leave sensitive patient information vulnerable. By methodically exploring a variety of algorithms and hyperparameters, we showed that our observation holds in the general case. Furthermore, we demonstrated that it is easier to reassociate sensitive information with rare names compared with common ones. Finally, we argue that, given our results, data holders and providers should explore whether other paradigms, such as PHI replacement, are more successful in securing sensitive information when compared with PHI removal.

### Background

#### Clinical Word Embeddings

Word embeddings (ie, *word vectors* or *distributed representations*) are dense numeric vectors used to represent words. Many word embedding techniques fall into one of two categories: low-rank approximations of co-occurrence matrices [4,9] and those created using shallow neural networks using contextual information [10]. There is also a recent and growing body of embedding models employing deeper neural networks to create contextual word embeddings, which vary depending on the surrounding context [11,12].

Inspired by the distributional hypothesis [13], word embeddings trained on health care data are strongly correlated with human-annotated word similarity metrics for medical terms [2], although their performance on clinical classification tasks is strongly dependent on the quality, size, and type of data from which they are created [14]. In fact, embeddings created from clinically related data (eg, clinical notes and biomedical text, such as a collection of all PubMed Central articles and PubMed abstracts), often performed better than, and never performed worse than, unspecialized corpora [2].

Until recently, there had been no publicly released embeddings trained on clinical data [15-18]. However, some newly released embeddings [15-18] are trained using contextual word embedding models on MIMIC, which itself uses PHI removal to abide by HIPAA regulations. Our work demonstrates how, if no additional security measures are taken, then traditional (noncontextual) models may be compromised. More work is required to assess whether our findings hold for the four new models as well (ie, contextual word embeddings) [15-18].

#### Privacy of Clinical Notes

There are 3 main approaches to protect the privacy of patients: dictionary-based, statistical, and hybrid approaches. Dictionary-based methods often use large wordlists or predefined regular expressions to locate private information in the text [6,19]. Statistical methods, often more robust than dictionary-based approaches [20], use models such as recurrent neural networks [7] to automatically detect private information. Hybrid methods combine the two approaches to compensate for their respective weaknesses [21]. No matter the method used to detect PHI, once it is detected, there are two ways to secure the data: PHI removal and PHI replacement.

#### Personal Health Information Removal

In PHI removal, sensitive information is located in text via a specialized search algorithm and then is either deleted or replaced with an informative deidentification tag (eg, all names are replaced with [*NAME*]). Although simple and common, this approach is not secure and can be easily exploited. Given that no PHI search algorithm is perfect, as a data set increases in size, it becomes increasingly certain that some PHI will be missed. Thus, if clinical notes are shared in a text format after this technique is used for deidentification, a malicious party can uncover names missed by the algorithm by manually inspecting the data. We demonstrate later that word embeddings created from such data are also vulnerable to similar exploits.

#### Personal Health Information Replacement

In PHI replacement, sensitive information, once located within the text by the search algorithm, is replaced with other information of the same type; for example, names can be randomly replaced with other names. This approach is more secure than PHI removal as it obscures instances where the PHI detection algorithm has failed and thus provides the data-curator with plausible deniability for any specific record.

We advocate that HCPs and data providers employ this paradigm because, if done correctly, it is much harder to exploit and thus reduces the risk to patient privacy. It is also a simple and effective way to protect against the exploitation of word embeddings that we demonstrated in this work.

## Methods

### Data

In our experiments, we used consultation notes. In Multimedia Appendix 1, we demonstrate how these findings are reproducible with an experiment performed with a selected subset of Wikipedia pages. We made the latter publicly available alongside the code. For all texts, we removed all punctuation and numeric characters, and we lowercased all text but performed no lemmatization, tokenization, or any other preprocessing.

We used consultation notes provided to the authors by ICES (formerly known as the Institute for Clinical Evaluative Sciences) under data sharing agreements with physicians for the purposes of evaluation and research. Consultation notes are written by specialist physicians and other health care consultants to a patient’s family physician. They describe the tests performed, results observed, and other details that the specialist physician or health care consultant considers relevant. We compiled patients' consultation notes and all their prescribed diagnostic codes that are indicative of suspected diagnoses and ordered tests, and are therefore sensitive health information that must not be connected to patient identities. The billing codes table includes text fields describing each code in 1 to 3 words, for example, *colon screening*. These data sets are linked using unique encoded identifiers and analyzed using ICES.

Although this work is conducted at ICES, ICES does not grant its research affiliates (including the authors of this paper) access to *true* patient names, but replaces them in the manner described earlier (PHI replacement), using a semimanual, dictionary-based masking process to consistently replace each true name with a randomly chosen fake name. We used heuristics to detect names in the notes. More concretely, we looked for semistructured notes that have *Name: str1, …, strN* (representing a series of alphabetical tokens separated by commas followed by a semicolon) to indicate the presence of a name. The heuristic is not 100% accurate, which is why, in Multimedia Appendix 2, we can provide only an estimate of how many true names exist by manually analyzing a randomly sampled set.

**Figure 1:**
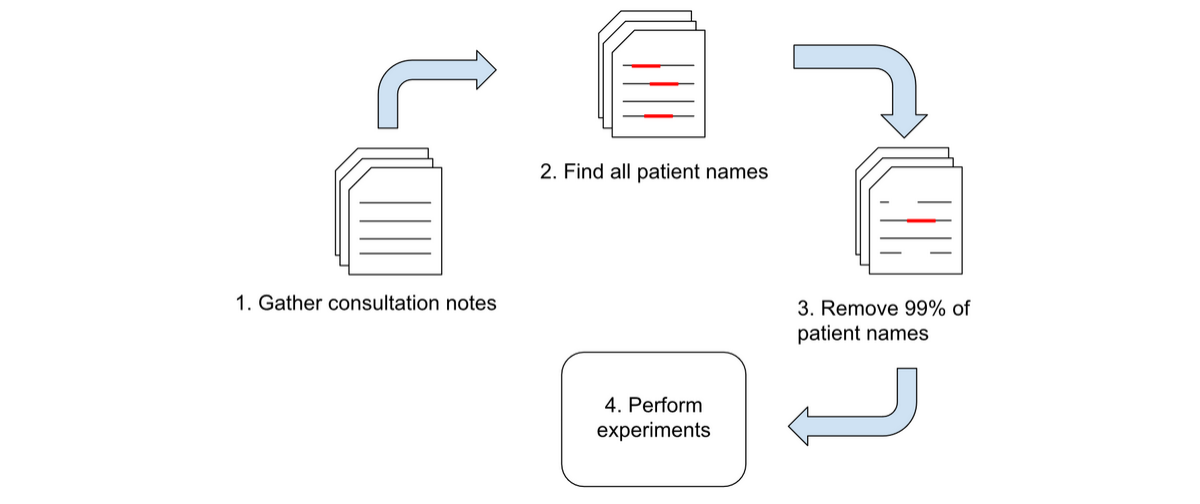
Process flow for gathering and preparing the clinical notes for embedding generation and experimentation.

We perform our experiments on clinical consultation notes for which we can locate the associated fake patient name. For our experiments, we treat the fake names as if they were the *true* names and removed 99% of them, thus emulating current PHI removal algorithms [7]. This protected data set is then used as the first step of our experiments, as shown in Figure 1. Detailed information regarding the data is provided in Multimedia Appendix 2.

### Experiments

#### Experimental Hypothesis

The intuition behind reidentifying patient information solely from word embeddings stems from the *distributional hypothesis* [13]—that words appearing in similar contexts tend to have similar meanings and therefore have closer vector representations than other words. Knowing this, we expect differences between both:

1. The average distance between the tokens that make up a person’s name, compared with tokens from different names.
2. The average distance in vector space between a person's name and their diagnoses (referred to as the in-group), compared with the average distance between their name and those diagnoses with which they are not associated (referred to as the out-group.

If there is a large enough distance between a person's in-group and out-group, then this observation could be used to extract sensitive information thought to have been hidden by the unordered nature of embeddings. In the following sections, we validated this hypothesis empirically.

#### Experiment 1: Name Reconstruction Experiment

**Figure 2:**
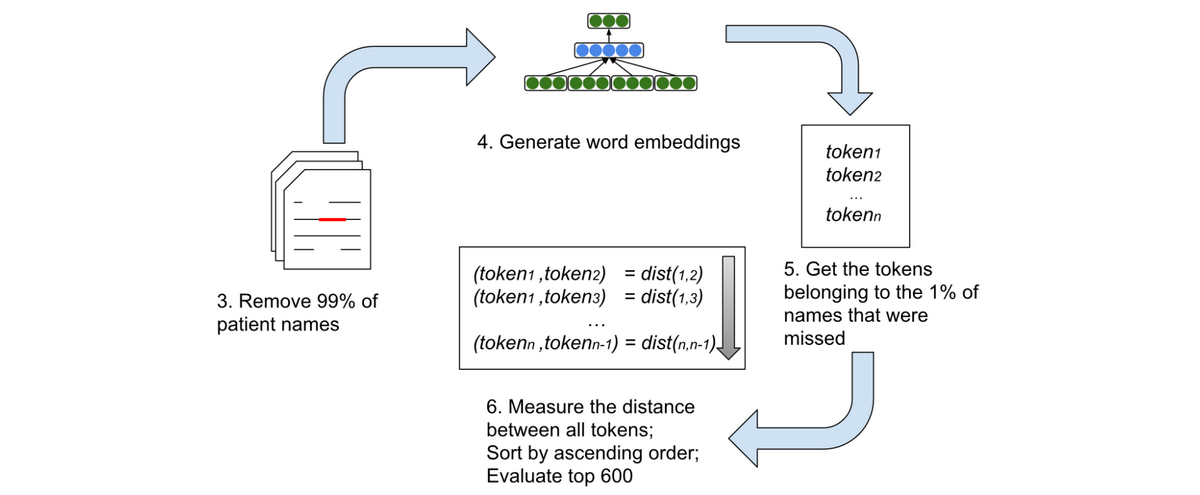
Process flow for generating word embeddings and performing the name reconstruction experiment.

In the first experiment, we tested whether it is possible to reconstruct true name pairs simply from a list of individual name tokens. Figure 2 presents the steps of this experiment, picking up from the last step of Figure 1.

A list of individual name tokens, corresponding to the fifth step in Figure 2, is easily generated by manual exploration of the words. However, as we left 1% of the names, to emulate the imperfect deidentification algorithms, we knew all the tokens (ie, the 1% of name tokens purposefully left in place).

We performed this experiment on our consultation notes data set, where over 99% of names were removed to emulate a PHI removal approach and only 1054 unique name tokens (from 650 full names) remained in the text.

We performed our experiment with 3 commonly used traditional word embedding algorithms (CBOW, Skipgram, and GloVe) for clinical prediction and modeling tasks. For each, we tested a variety of hyperparameters. Where a specific hyperparameter is not explicitly mentioned, we used the default hyperparameter of the training model, which can be found in Multimedia Appendix 2.

However, for the sixth step, an attacker would not know how many full names were in the data set. If we assume that each name is composed of 2 tokens and none of the names share any name tokens; we would expect the number of complete names to be half the number of name tokens (ie, 1052/2 complete names). Relaxing both assumptions increases the expected names. Given name tokens *A* and *B*, we considered a name to exist if either 〈*A,B*〉 or 〈*B,A*〉 exist as names (ie, ignoring ordering). On this data set, we created many word embedding models (n=88) with a wide set of hyperparameters (ie, model specifications) that included variations in the distance metric (cosine or cityblock) and context window size.

#### Experiment 2: Name-Diagnostic Code Association Experiment

In this section, we explored the second part of our hypothesis: is there a difference between the average distance in vector space between a person's name and their diagnoses (their *in-group*) compared with the average distance between their name and those diagnoses with which they are *not* associated (their *out-group*)?

For this experiment, we used the same data and tested the properties of the same word embedding algorithms for various hyperparameters, as in the last experiment. We first define a patient's name vector as the average of the vectors of its components (ie*,* first, last, and possibly middle names). Here, *numtoken* is the number of space-separated tokens in a string and is the vector representation of the *i*th token of the name:


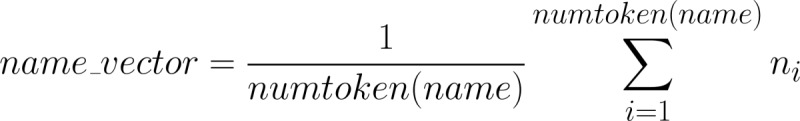


Second, we defined the in-group *d_in_* as the set of diagnoses for *name* and the out-group *d_out_*, as all other diagnoses, with *d_i_* representing any individual diagnosis. The average distance for each of these groups from their respective names are referred to as *in_group* and *out_group,* respectively:


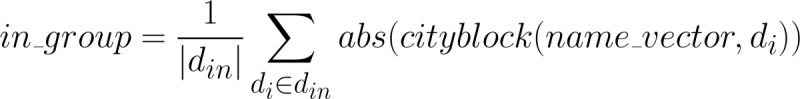



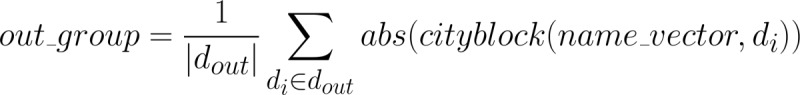


We presented the results using the *cityblock* distance (ie, the Manhattan distance) instead of the cosine distance because it performs better at this task (by uncovering more information), and past work has shown that the vector magnitude (ie*,* the sum of all dimensions) is affected by the number of times that the word occurs in the corpus [22]. However, our experiments were performed using the cosine distance metric as well, and complete results can be found in Multimedia Appendix 2.

Initially, we explored the raw data (ie, without any deidentification algorithm) by plotting the difference between the in- and out-groups for names that occur below different frequency thresholds. A name is below the threshold if the average counts of its components are below that threshold. For example, if “James” occurs 201 times in the corpus and “Qwerty” appears twice, then “James Qwerty” is below an arbitrary threshold of 200 (101.5<200).

**Figure 3:**
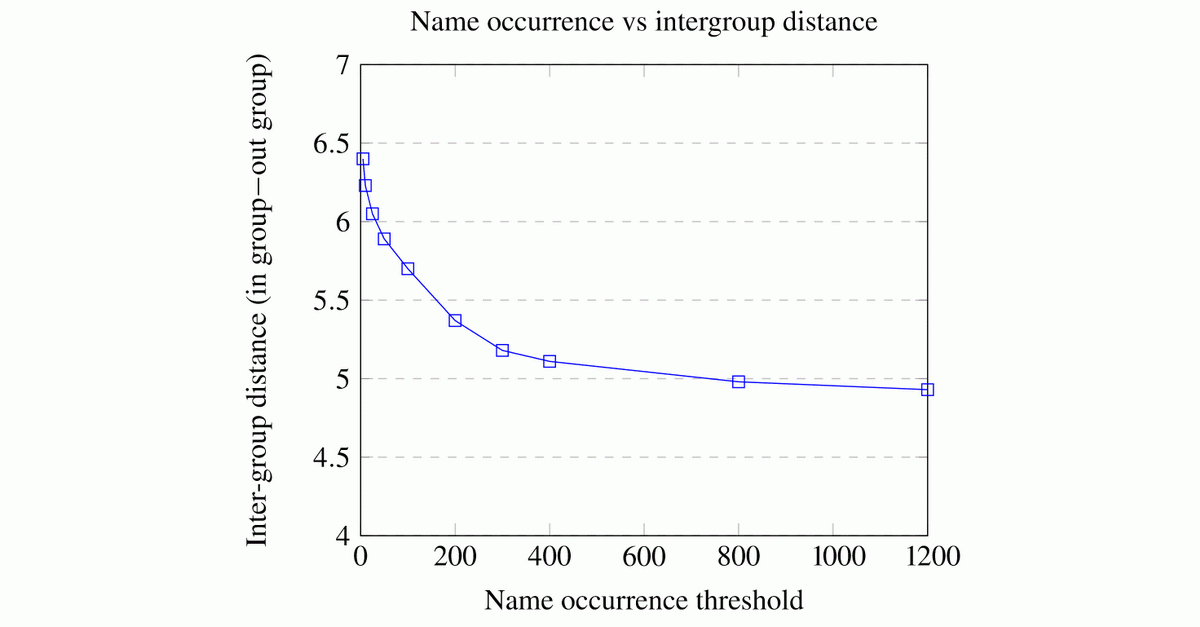
Relationship between frequency of name occurrence and the average difference between the in-group and out-group for patients. This graph is generated from an experiment run on a GloVe model with a dimension of 100, window of 10, learning rate of 0.05, minimum occurrence of 1, and alpha of .75.

Figure 3 shows that the more frequently a name occurs, the smaller the difference between the in-groups and out-groups. Nonetheless, the difference is still pronounced when all names are considered, with the lowest value being just under 5. Surprisingly, against our intuition, the in-group is larger than the out-group. We saw this result consistently throughout our testing described in the following sections.

#### Statistical Testing

**Figure 4:**
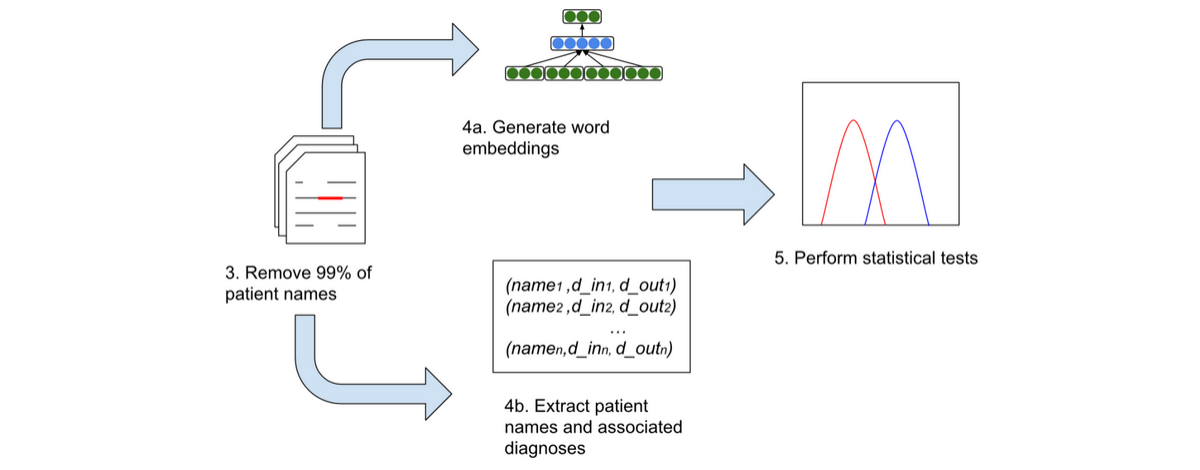
Process flow for generating word embeddings and performing statistical testing. For population-level statistical testing, we performed a Wilcoxon signed-rank test, and for patient-level statistical testing, we calculated empirical *P* values using 1000 randomly generated permutations.

Given our initial observation that, on raw data, there is a difference between in- and out-groups on the population level on raw data, we now examine if the observed differences are statistically significant at both the population and patient levels for various embedding algorithms and hyperparameters on the deidentified data set (ie, 99% of names have been removed to emulate an optimum real-life data sharing scenario). A diagram of the experimental process is shown in Figure 4.

#### Experiment 2a: Population-Level Statistical Testing

In this experiment, we aimed to determine whether the difference between the in- and out-groups on the population level is statistically significant.

Here, as with all the clinical text experiments, the embedding model is trained using all consultation notes after 99% of the names have been removed. Using the same setup as in the previous section to obtain distances between in- and out-groups, we used the Wilcoxon signed-rank test to compare the pairings of in- and out-groups for each name on the population level. The Wilcoxon signed-rank test is nonparametric and, unlike the paired Student two-tailed *t* test, makes no assumptions regarding normality.

This experiment is performed for various embedding algorithms, distance metrics, and hyperparameter ranges.

#### Experiment 2b: Patient-Level Statistical Testing

Here, we explored whether there is a statistically significant difference between the in- and out-groups for each patient, which would indicate that an individual patient is at risk of having their diagnostic code uncovered.

In this experiment, we compared the average difference between a patient’s in-group and the out-group. Although each comparison will result in a *P* value for each patient, for brevity and privacy, we do not report the per patient analysis of the ICES data, but instead report the number of patients for which the difference is significant after correcting for multiple comparisons. To determine statistical significance at the patient level, we calculated empirical *P* values by randomly sampling in- and out-groups generated using 1000 permutations of the same size from the same data set.

We experimented with various embedding algorithms, distance metrics, and hyperparameter ranges.

#### Experiment 3: Scenario Simulation

In this experiment, we performed a hypothetical attack to examine whether the results of the previous 2 experiments demonstrate an actionable level of risk. Assuming the role of an attacker who has access only to released embeddings built from doctor-patient consultation notes that have been secured by using PHI removal, we showed how we are able to associate name tokens that were missed by PHI removal to arrive at a list of complete patient names and that we are able to associate these names with some target diagnoses.

For this hypothetical scenario, we used the same data and tested the properties of the same word embedding algorithms for various hyperparameters as in the last experiment.

The attack is as follows:

1. Identify a list of target diagnoses that we wish to attribute to patients. As an example, we considered the following set of diagnoses: constipation, diarrhea, vaginitis, sexual dysfunction, urinary infection, herpes genitalis, dementia, anorexia, alcoholism, threatened abortion, and AIDS.
2. For each name, calculate the 5 diagnoses that are farthest from the name.
3. Using these 5 diagnoses as the basis for prediction, we calculated Top-1 (A@1) and Top-5 (A@5) accuracy.

To ensure that our results are not an artifact of the selected diagnoses, we repeated the above experiment 1000 times for each tested hyperparameter, randomly selecting 30 target diagnoses. To be as stringent as possible, we chose from diagnoses that appeared at least 10 times in the data (which likely will result in a pessimistic bias, as demonstrated in Multimedia Appendix 2).

## Results

### Experiment 1: Name Reconstruction Experiment

The results of this experiment demonstrate that it is possible to reconstruct true name pairs simply from a list of individual name tokens and their respective embeddings.

**Figure 5:**
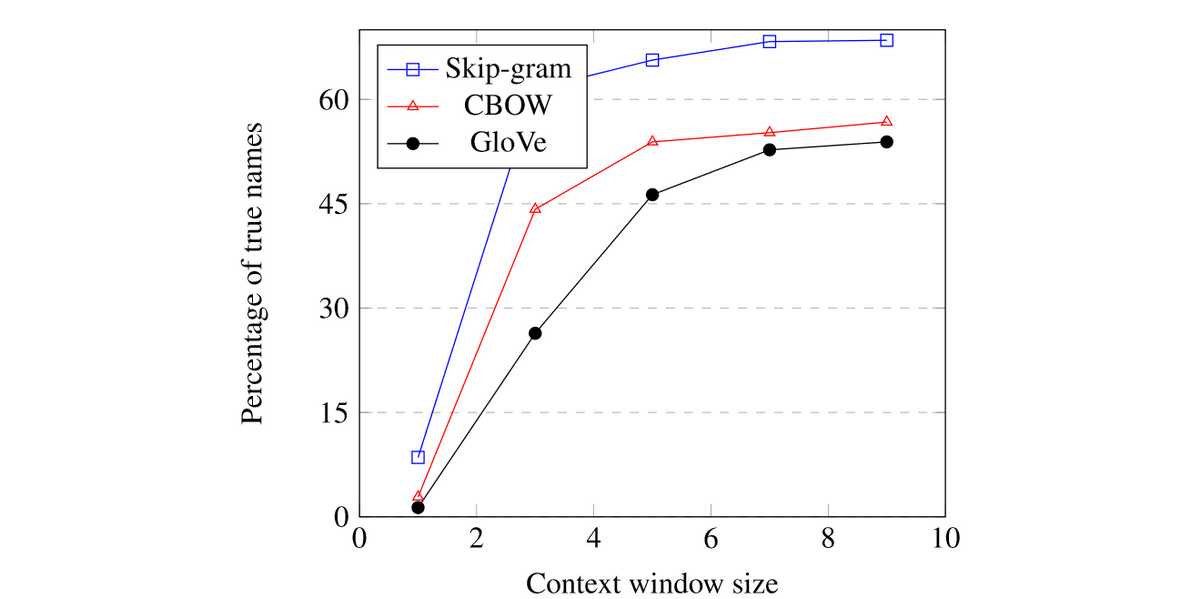
Visual representation of the percentage of paired names belonging to true names from the first 600 paired tokens when sorted in ascending order.

In this section, we present the results for various context window sizes, an expected name list of size 600, and a cosine distance metric. We observed that up to 68.5% (411/600) of the paired tokens come from true names, as shown in Table 1 and Figure 5. As there are over 170,000 name-pair combinations, these embeddings clearly carry patient information that can be identified, thus affirming our hypothesis. The complete results for other hyperparameters, the number of names expected, and the cityblock distance metric are presented in Multimedia Appendix 2.

**Table 1:** The number and percentage of paired tokens that are part of true names as a function of context window size, using the cosine distance metric of the first 600 paired tokens sorted in ascending order.

Context window size	Skipgram names, n (%)	CBOW^a^ names, n (%)	GLoVe^b^ names, n (%)
1	51 (8.5)	17 (2.8)	8 (1.3)^c^
3	369 (61.5)	265 (44.2)	158 (26.3)
5	393 (65.6)	323 (53.8)	278 (46.3)
7	410 (68.3)	331 (55.2)	317 (52.8)
9	411 (68.5)	340 (56.7)	323 (53.8)

^a^CBOW: Continuous Bag of Words.

^b^GLoVe: Global Vectors.

^c^Result not significant after correcting for multiple comparisons using the Holm-Bonferroni correction.

### Experiment 2: Name-Diagnostic Code Association Experiment

#### Experiment 2a: Population-Level Statistical Testing

**Figure 6:**
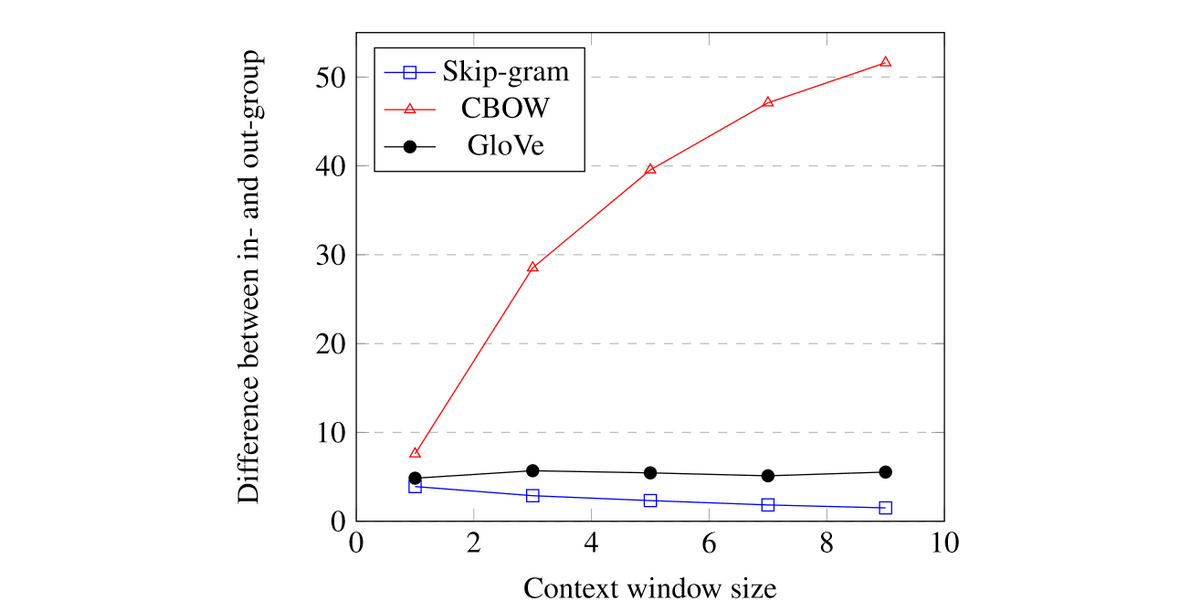
Visualization of the difference between the in-group and the out-group as a function of context window size for various word embedding algorithms using the cityblock distance metric.

The results of this experiment indicate that, at the population level, the average difference between the in- and out-groups per patient is statistically significant. Table 2 and Figure 6 show the results for various embedding algorithms, varying context window sizes, and a cityblock distance metric. The complete results for other hyperparameters, other distance measures, and absolute distances are shown in Multimedia Appendix 2.

**Table 2:** Difference between the in-group and out-group as a function of context window size for various word embedding algorithms using the cityblock distance metric. The differences are relative distances between word embedding vectors in an n-dimensional space.

Context window size^a^	Skipgram difference	CBOW^b^ difference	GLoVe^c^ difference
1	3.91	7.59	4.85
3	2.88	28.53	5.69
5	2.33	39.55	5.45
7	1.84	47.10	5.12
9	1.51	51.61	5.54

^a^All differences were statistically significant after correcting for multiple comparisons.

^b^CBOW: Continuous Bag of Words.

^c^GLoVe: Global Vectors.

Given our selected hyperparameters, we observed that for all sizes tested and for all embedding techniques, the difference between the in- and out-groups on the population level was statistically significant with *P*<.001 calculated using the Wilcoxon test, after correcting for multiple comparisons using the Holm-Bonferroni correction [23]. The Holm-Bonferroni correction is a sequentially rejective procedure for correcting multiple comparisons that keeps the family-wise type I error bounded. Figure 6 shows that the difference between the in-group and out-group decreases for embeddings created with the Skipgram algorithm as the context window increases. Conversely, the difference grows for CBOW, while it remains relatively stable for all GloVe models.

#### Experiment 2b: Patient-Level Statistical Testing

Building on our previous observations, the results of this experiment indicate that, at the patient level, for a percentage of examined patients (up to 449/638, 70.4%), the average difference between in- and out-groups per patient is statistically significant.

**Figure 7:**
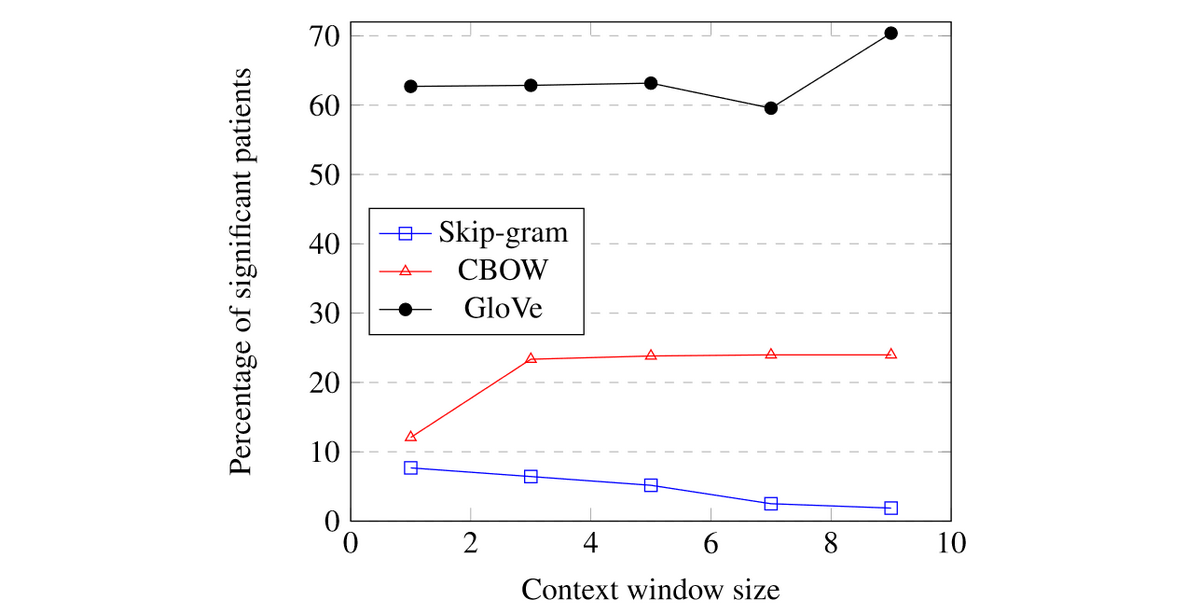
Visualization of the percentage of patients who have a significant difference between their in- and out-groups as a function of context window size for multiple word embedding algorithms using the cityblock distance metric.

Table 3 and Figure 7 show the results for various embedding algorithms, varying context window sizes, and a cityblock distance metric. The complete results for other hyperparameters, other distance measures, and absolute distances are shown in Multimedia Appendix 2.

**Table 3:** The percentage of patients whose diagnoses are identifiable due to a statistically significant difference between the in-group and out-group as a function of context window size for various word embedding algorithms using the cityblock distance metric.

Size	Skipgram patients, %	CBOW^a^ patients, %	GLoVe^b^ patients, %
1	49 (7.7)	77 (12.1)	400 (62.7)
3	41 (6.4)	149 (23.4)	401 (62.8)
5	33 (5.2)	152 (23.8)	403 (63.2)
7	16 (2.5)	153 (24.0)	380 (59.6)
9	12 (1.9)	153 (24.0)	449 (70.4)

^a^CBOW: Continuous Bag of Words.

^b^GLoVe: Global Vectors.

Table 3 presents the patient-level analysis for different context window sizes. As shown in Figure 7, using the CBOW algorithm, an increasing window size initially correlates positively with the number of vulnerable patients, defined as having a significant difference between the in-group and out-group. The opposite trend is observed for the Skipgram model. Context window size does not appear to have an effect on word embeddings created using GloVe, as the number of patients remains relatively stable.

### Experiment 3: Scenario Simulation

Having demonstrated that the difference between in- and out-groups is statistically significant, in this section, we showed that our hypothetical attack results in an actionable level of risk, that is, an attacker who has access only to released embeddings built from doctor-patient consultation notes that have been secured by using PHI removal may be able to arrive at a list of complete patient names, and associate these names with target diagnoses.

We observed that for our chosen target diagnoses (ie, constipation, diarrhea, vaginitis, sexual dysfunction, urinary infection, herpes genitalis, dementia, anorexia, alcoholism, threatened abortion, and AIDS) our approach out performs the majority baseline for both top 1 (A@1) and top 5 (A@5) accuracy of 0.00 and 0.70, respectively, Multimedia Appendix 2 (top *n* rate is the fraction of examples for which the correct label is among the *n* labels considered most probable by the model). The complete results for all hyperparameters as well as both distance metrics are presented in Multimedia Appendix 2.

We observed similar results when the above experiment was repeated 1000 times for each tested hyperparameter, randomly selecting 30 target diagnoses. Table 4 shows how often our attacker’s approach surpasses the baseline of choosing the majority diagnoses for both top-1 and top-5 accuracies. We show that we can consistently beat strong baselines, although the highest top-1 and top-5 accuracies are modest at 0.08 and 0.15, respectively. The complete results for all hyperparameters as well as both distance metrics are presented in Multimedia Appendix 2.

**Table 4:** The percentage of times using a word embedding–based attack beats the majority baseline for A@1 and A@5 for various context window sizes over 1000 random diagnosis selections.

Context window size^a^	Skipgram A@1, A@5	CBOW^b^ A@1, A@5	GLoVe^c^ A@1, A@5
1	55.8, 56.7	61.8, 61.8	55.4, 56.9
3	55.6, 53.1	51.2, 52.6	60.5, 59.5
5	57.4, 55.6	53.6, 54.5	59.4, 57.2
7	57.4, 53.5	54.6, 53.9	55.9, 54.0
9	57.2, 53.2	53.7, 51.2	60.6, 56.7

^a^We observed that the majority baseline is surpassed consistently and up to 60% of the time.

^b^CBOW: Continuous Bag of Words.

^c^GLoVe: Global Vectors.

## Discussion

### Principal Findings

In this work, we have shown the following:

- There is a statistically significant difference between the distance of patients’ in- and out-groups at the population level.
- For many individual patients, the difference between their personal in-group and out-group is also statistically significant.
- A malicious actor working only with word embeddings may identify full names occurring in the training corpus of the embeddings as well as sensitive attributes associated with these names.

### Limitations

We explored the induced privacy (or lack of privacy) of embeddings created from medical notes. We empirically highlighted the security risks of sharing clinically sourced word embeddings. Although their nature does serve to obfuscate information, we have shown that it is still possible to connect PHI to names from word embeddings secured using PHI removal. There is much variation in the risks observed in this work, which are dependent on imperfect deidentification algorithms and very skilled attackers. The actual risk to patient information, while nonzero, remains small and dependent on many variables such as the attack strategy, deidentification method, and embedding algorithm. We therefore advocate for more research to see whether the adoption of PHI replacement would better secure released embeddings. In addition to deidentification methods (where more research needs to be done), appropriate controls on who can access the anonymized data and oversight of these data are also recommended.

### Conclusion

We have focused on the reidentification of names and their association with diagnostic codes, although other sensitive PHIs may also be vulnerable. We demonstrated how sharing word embeddings trained on clinical notes that have been protected using only PHI removal is not safe, as any PHI missed by the algorithm will remain in its original context. The risk of obtaining sensitive information from embeddings can be diminished by applying the anonymization methods of PHI replacement on the clinical notes before training the embedding, that is, when all known PHIs have been randomly shuffled, it becomes much more difficult (but not impossible) to determine which names in the data set belong to true patients, as the names that are shuffled together will behave in a manner similar to true names that have been missed. Such embeddings can theoretically still be at risk if an attacker is able to determine how to differentiate between fake and true names. However, this would mitigate the methods of attack described in this work, thereby making the created embeddings more secure. Alternatively, noise can be added to the generated embedding model to induce privacy and reduce risk. This risk reduction is, naturally, relative to the amount of noise added, and determining the exact amount of noise without distorting the signal or degrading performance is the subject of future research.

Regarding reassociating name tokens or associating names with diagnostic codes, Skipgram is least effective at preserving privacy, followed by CBOW and GloVe. However, when examining the number of statistically significant differences, we observe the opposite ranking. Although many sentence- and text-classification tasks observe little difference in downstream performance between these 3 algorithms, past work [5] has demonstrated differences in the ability of these algorithms to represent words. For example, Skipgram can perform better than CBOW for more frequent words [5], possibly explaining the difference in modeling names (which are infrequent in our vocabulary).

As expected, tokens from the same complete name have closer vector representations. However, despite our intuition, we find that the in-group is surprisingly larger than the out-group. That is, the average distance between a name and a diagnosis is larger if the person with that name has a diagnosis. This was consistent among all parameters tested, and among all 3 embedding models. This was also observed in our novel data set. This was perplexing because our expectation of word embeddings informs us that words that occur in similar contexts should be closer together, and in-group diagnoses are often in the same note as the name, while out-group diagnoses are not in the note at all. Even though the name and diagnosis tokens may not co-occur directly, as they would gravitate to words that co-occur with both, this would result in the names and in-group diagnoses being closer. Although a deeper theoretical investigation remains to be conducted, we hypothesize that this may be due to interaction effects within the contexts; names are quite tightly clustered together, and they rarely occur in the same context window of the diagnosis with which they are associated. It may be that these other names draw the *common* diagnoses closer, as they occur with more names, in turn leaving the less common, but relevant, diagnoses further from the *name* cluster. This requires further research.

## Appendix

Multimedia Appendix 1 Wikipedia Appendix.

Multimedia Appendix 2 ICES Appendix.

## Abbreviations

CBOW: Continuous Bag of Words
GLoVe: Global Vectors
HCP: health care provider
HIPAA: Health Insurance Portability and Accountability Act
MIMIC: Multiparameter Intelligence Monitoring in Intensive Care
NLP: natural language processing
PHI: personal health information
