# Supplementary material for: Exploring the Privacy-Preserving Properties of Word Embeddings: Algorithmic Validation Study
Source: J Med Internet Res. 2020 Jul 15;22(7):e18055. doi: 10.2196/18055 (PMC7391163; doi:10.2196/18055)
Supplement: Multimedia Appendix 1 [file jmir_v22i7e18055_app1.docx]

**APPENDIX 1 – Wikipedia Experiment**

| **Number of pages** | 19,686 |
| --- | --- |
| **Words per page** | 251 |

**Appendix Table 1.** Characteristics of Wikipedia dataset.

**Scenario Simulation on Wikipedia Data**

While data-sharing agreements prohibit ICES from making our clinical dataset publicly available, access may be granted to those who meet pre-specified criteria for confidential access, available at www.ices.on.ca/DAS.

In this section, however, we aim to replicate the results of the ICES experiments with a novel dataset that can be publicly released, and to answer questions that were not possible with the ICES dataset. To model patient data in a dataset that can be publicly released without privacy concerns, we built a synthetic dataset composed of approximately 20,000 biographies of politicians by scraping the “Politician” category in Wikipedia. Each biography can be regarded as a model “clinical note” about its subject, upon which we can experiment. We “de-identified” our dataset of 20,000 politicians’ biographies by removing 99% of the names, leaving behind only 200 names as if they had been missed by a state-of-the-art PHI removal algorithm. We considered only space-separated tokens in the title as “names”, choosing to ignore other possible names. The embedding model used for all the following experiments is a CBOW word embedding model with a context window of 5 trained on this “secured” version of the 20,000 biographies.

First, we show that it is possible to reconstruct entire name pairs simply from a list of individual name tokens. To do this, we measure the cosine distance between each pairing of individual name tokens and sort the resulting list in ascending order. We observe that the first 10 paired tokens are correctly paired and that, of the top 50 paired tokens, 36 (72%) are correctly paired. This result indicates that it is possible to reconstruct full names (or parts of full names) simply from the embedding model itself. This lowers the cost of an attack by making it easier to identify people in a released model.

Second, we attempt to determine the nationality of each politician using only the word embedding model. Here, nationality is analogous to a diagnostic code in the previous experiment. As observed before, the in-group has a higher distance than the out-group with average distances of 64.4 and 51.0, respectively. Performing the same hypothetical attack as Experiment 3 results in top-1 and top-5 accuracies of 9.8% and 21.3% respectively, with a large increase in top-1 accuracy and a maintained performance in the top-5 accuracy.

Finally, we manually explore the nearest neighbors of name vectors as well as words most likely to be predicted as the focal word given the name as context. This analysis is done qualitatively for the first 5 named pairs that were correctly paired. The full list of predicted words for each name is presented in uploaded as part of the supplemental materials (as a Jupyter notebook). We observe that such information, although quite noisy, contains terms relevant for some names (including the birthplace, and “*guilty, tribunal, murder*”). This information could be used by a malicious actor to gain more information about a patient.
