# Supplementary material for: Exploring the Privacy-Preserving Properties of Word Embeddings: Algorithmic Validation Study
Source: J Med Internet Res. 2020 Jul 15;22(7):e18055. doi: 10.2196/18055 (PMC7391163; doi:10.2196/18055)
Supplement: Multimedia Appendix 2 [file jmir_v22i7e18055_app2.docx]

**APPENDIX 2 – ICES Experiments**

| **Number of patients** | 89,990 |
| --- | --- |
| **Notes with a name** | 402,793 |
| **Words per note** | Min = 1; avg = 212; max = 10,473 |
| **Word count** | 84,278,374 |
| **Unique word count** | 366,977 |
| **Unique code words** | 9,094 |
| **Code words per patient** | Min = 1, avg = 21, max = 163 |

**Appendix Table 1.** Characteristics of ICES dataset.

**Word Embedding Model Parameters**

For CBOW and Skipgram the initial parameters are: Window Size = 5, Learning Rate = 0.025, min_count = 1, epochs = 25.

For GloVe the parameters are: Window Size = 10, Learning Rate = 0.05, Alpha = 0.75, min_count = 1, epochs = 35.

**Frequency of Diagnostic Codes**

| **Number of patients with Diagnostic Code** | **Number of Diagnostic Codes** |
| --- | --- |
| constipation | 4146 |
| diarrhea | 9064 |
| vaginitis | 6770 |
| sexual dysfunction | 1043 |
| urinary infection | 1174 |
| herpes genitalis | 172 |
| dementia | 1851 |
| anorexia | 466 |
| alcoholism | 1581 |
| threatened abortion | 546 |
| AIDS | 24 |

**Appendix Table 2.** Frequency of diagnostic codes used in the hypothetical scenario presented in the paper.

**Analysis of Diagnostic Code**

| **Number of patients with Diagnostic Code** | **Number of Diagnostic Codes** |
| --- | --- |
| **1** | 9707 |
| **2** | 1904 |
| **3** | 703 |
| **4** | 454 |
| **5** | 291 |
| **6** | 239 |
| **7** | 182 |
| **8** | 179 |
| **9** | 129 |
| **10–50** | 1503 |
| **50–300** | 870 |
| **300–Max (31472)** | 598 |

**Appendix Table 3.** Number of diagnostic codes that appear for varying number of patients.

**Studying effect of Frequency**

In this section, we study the effect of diagnostic code frequency on statistical significance. In particular, we attempt to see whether the statistically significant distances observed in the experiments are due to the long-tail in the dataset (i.e., the singly occurring diagnoses that make up the majority of the dataset). To this end, we recreate the experiment at both the population and the patient level while excluding diagnoses that occur fewer than 5 times and fewer than 10 times (Table 4).

| *Context Window Size – Cityblock Measure – Diagnostic Codes (ALL)* | |
| --- | --- |
| \| **Size** \| **Skipgram %patients** \| **CBOW %patients** \| **GLoVe**  **%patients** \| \| --- \| --- \| --- \| --- \| \| 1 \| 7.7 \| 12.1 \| 62.7 \| \| 3 \| 6.4 \| 23.4 \| 62.8 \| \| 5 \| 5.2 \| 23.8 \| 63.1 \| \| 7 \| 2.5 \| 24.0 \| 59.6 \| \| 9 \| 1.9 \| 24.0 \| 70.4 \|   (a) | \| **Size** \| **Skipgram distance** \| **CBOW distance** \| **GLoVe**  **distance** \| \| --- \| --- \| --- \| --- \| \| 1 \| 3.91 \| 7.59 \| 4.85 \| \| 3 \| 2.88 \| 28.53 \| 5.69 \| \| 5 \| 2.33 \| 39.55 \| 5.45 \| \| 7 \| 1.84 \| 47.10 \| 5.12 \| \| 9 \| 1.51 \| 51.61 \| 5.54 \|   (b) |
| *Context Window Size – Cityblock Measure – Diagnostic Codes (N ≥ 5)* | |
| \| **Size** \| **Skipgram %patients** \| **CBOW %patients** \| **GLoVe**  **%patients** \| \| --- \| --- \| --- \| --- \| \| 1 \| 0.9 \| 3.8 \| 51.4 \| \| 3 \| 1.2 \| 3.9 \| 49.5 \| \| 5 \| 1.1 \| 3.9 \| 48.7 \| \| 7 \| 0.6 \| 4.2 \| 46.2 \| \| 9 \| 0.2 \| 4.2 \| 59.2 \|   (c) | \| **Size** \| **Skipgram distance** \| **CBOW distance** \| **GLoVe**  **distance** \| \| --- \| --- \| --- \| --- \| \| 1 \| 1.86 \| 3.73 \| 3.04 \| \| 3 \| 1.34 \| 14.58 \| 3.40 \| \| 5 \| 0.94 \| 20.25 \| 3.29 \| \| 7 \| 0.71 \| 23.71 \| 2.87 \| \| 9 \| 0.50 \| 26.18 \| 3.22 \|   (d) |
| *Context Window Size – Cityblock Measure – Diagnostic Codes (N ≥ 10)* | |
| \| **Size** \| **Skipgram %patients** \| **CBOW %patients** \| **GLoVe**  **%patients** \| \| --- \| --- \| --- \| --- \| \| 1 \| 1.6 \| 2.5 \| 45.4 \| \| 3 \| 1.4 \| 1.6 \| 42.5 \| \| 5 \| 0.8 \| 2.0 \| 43.4 \| \| 7 \| 0.5 \| 1.4 \| 39.3 \| \| 9 \| 0.3 \| 1.6 \| 54.2 \|   (e) | \| **Size** \| **Skipgram distance** \| **CBOW distance** \| **GLoVe**  **distance** \| \| --- \| --- \| --- \| --- \| \| 1 \| 1.51 \| 3.03 \| 2.47 \| \| 3 \| 1.15 \| 11.58 \| 2.81 \| \| 5 \| 0.79 \| 16.10 \| 2.73 \| \| 7 \| 0.61 \| 18.65 \| 2.31 \| \| 9 \| 0.43 \| 20.79 \| 2.67 \|   (f) |

**Appendix Table 4**. The percentage of patients whose diagnoses is identifiable due to a statistically significant difference distance between in-group and out-group as a function of various hyperparameter setting, using the cityblock measure. Sub-tables a) and b) consider all diagnostic codes. Sub-tables c) and d) consider diagnostic codes that occur at least 5 times across all patients. Sub-tables e) and f) consider diagnostic codes that occur at least 10 times across all patients. To determine statistical significance at the patient level, we calculated empirical *p*-values by randomly sampling the in- and out-groups generated using 1000 permutations of the same size from the same dataset. At the population level, we use the Wilcoxon signed-rank test to compare the pairings of in- and out-groups for each name. All presented distances are significant after correcting for multiple comparisons using Holm-Bonferroni correction.

We observe that the more restrictive we are with the diagnostic codes, the less statistical significance we observe at the patient level. The drop is especially apparent for the CBOW and Skipgram algorithms, while the GloVe models still reveal much about patients. However, on the population level, the in-group is still significantly higher than the out-group consistently for all three algorithms, Appendix Table 4 (b) (d) and (f). To demonstrate the “*actionable* *risk*” posed even by the most highly-restricted dataset, we randomly sample from the restricted set of diagnostic codes in the last scenario performed in the work where we play a hypothetical attacker.

We also explored whether the mean frequency of diagnostic codes from the in-group when compared to the mean frequency of diagnostic codes from the out-group was correlated to the difference between the in- and out-groups. We found that there is little to no correlation between the frequency of diagnostic codes in the in-group vs out-group and difference between in- and out-groups, with most comparisons lacking statistical significance, Appendix Table 5.

| *Context Window Size – Cityblock Measure – Diagnostic Codes (N ≥ 5)*   \| **Size** \| **Skipgram correlation** \| **CBOW correlation** \| **GLoVe**  **correlation** \| \| --- \| --- \| --- \| --- \| \| 1 \| –0.023 ^a^ \| 0.005 ^a^ \| 0.140 \| \| 3 \| –0.014 ^a^ \| 0.074 ^a^ \| 0.158 \| \| 5 \| –0.008 ^a^ \| 0.075 ^a^ \| 0.134 \| \| 7 \| –0.023 ^a^ \| 0.072 ^a^ \| 0.125 ^a^ \| \| 9 \| –0.041 ^a^ \| 0.062 ^a^ \| 0.158 \|   (a) | *Context Window Size – Cityblock Measure – Diagnostic Codes (N ≥ 10)*   \| **Size** \| **Skipgram correlation** \| **CBOW correlation** \| **GLoVe**  **correlation** \| \| --- \| --- \| --- \| --- \| \| 1 \| –0.026 ^a^ \| 0.001 ^a^ \| 0.136 \| \| 3 \| –0.016 ^a^ \| 0.074 ^a^ \| 0.155 \| \| 5 \| –0.010 ^a^ \| 0.073 ^a^ \| 0.131 \| \| 7 \| –0.021 ^a^ \| 0.072 ^a^ \| 0.122 ^a^ \| \| 9 \| –0.041 ^a^ \| 0.060 ^a^ \| 0.154 \|   (b) |
| --- | --- | --- | --- | --- | --- | --- | --- | --- | --- | --- | --- | --- | --- | --- | --- | --- | --- | --- | --- | --- | --- | --- | --- | --- | --- | --- | --- | --- | --- | --- | --- | --- | --- | --- | --- | --- | --- | --- | --- | --- | --- | --- | --- | --- | --- | --- | --- | --- | --- |

**Appendix Table 5**. Spearman’s rank correlation between in-group frequency and in- and out-group differences as a function of varying context window sizes for various word embedding algorithms using the cityblock distance for diagnostic codes that appear more at least (a) 5 times, (b) 10 times across all patients.. A superscript ‘a’ denotes lack of significance after correcting for multiple comparisons using the Holm-Bonferroni method. We see that there is little to no correlation between the two variables.

**Considering Effect-Size**

Appendix Table 4 demonstrated the statistical significance at the patient and population level. In this section we present the effect-size of the difference between the in- and out-groups both at the population and the patient level. The effect size, Appendix Equation 1, serves to communicate the magnitude of difference between two groups as opposed to the binary test of significance. Furthermore, unlike the statistical tests performed in the paper, effect size is independent of sample size.

$$Effect Size= \frac{\left[ mean of group1 \right]-[mean of group2]}{Standard Deviation}$$

**Appendix Equation 1**. Formulation of the effect size.

Appendix Table 6 presents the effect size when comparing the in- and out-groups for both the population level and the patient level. In this comparison, we only use diagnostic codes that occur over 10 times (as this was the most-restrictive setting observed in Appendix Table 4). We observe that at the population level we have large effect sizes for CBOW and GloVe, and medium to small effect sizes for Skipgram. At the patient level, we observe a small average effect size.

| *Context Window Size – Cityblock Measure – Population Level*   \| **Size** \| **Skipgram Effect Size** \| **CBOW Effect Size** \| **GLoVe**  **Effect Size** \| \| --- \| --- \| --- \| --- \| \| 1 \| 0.463 \| 0.684 \| 0.852 \| \| 3 \| 0.330 \| 0.911 \| 0.997 \| \| 5 \| 0.221 \| 0.911 \| 0.970 \| \| 7 \| 0.161 \| 0.905 \| 0.836 \| \| 9 \| 0.108 \| 0.912 \| 0.973 \|   (a) | *Context Window Size – Cityblock Measure – Patient Level*   \| **Size** \| **Skipgram Effect Size** \| **CBOW Effect Size** \| **GLoVe**  **Effect Size** \| \| --- \| --- \| --- \| --- \| \| 1 \| 0.237 \| 0.278 \| 0.293 \| \| 3 \| 0.211 \| 0.352 \| 0.372 \| \| 5 \| 0.146 \| 0.342 \| 0.365 \| \| 7 \| 0.117 \| 0.335 \| 0.310 \| \| 9 \| 0.078 \| 0.333 \| 0.367 \|   (a) |
| --- | --- | --- | --- | --- | --- | --- | --- | --- | --- | --- | --- | --- | --- | --- | --- | --- | --- | --- | --- | --- | --- | --- | --- | --- | --- | --- | --- | --- | --- | --- | --- | --- | --- | --- | --- | --- | --- | --- | --- | --- | --- | --- | --- | --- | --- | --- | --- | --- | --- |

**Appendix Table 6**. (a) Effect size comparing the in- vs out-group distances as a function of context window size for multiple word embedding algorithms using the cityblock distance measure at the population level. (b) Mean effect size comparing the in- vs out-group distances for each patient as a function of the context window size for multiple word embedding algorithms using the cityblock distance measure.

**Name Reconstruction Parameters**

For the name reconstruction experiments in the paper and full results in the appendix, we explore what percentage of the first 600 names sorted by ascending order are part of existing patient names. In this section, we explore the effect of choosing different limits. We can see that expanding the list of names which we look at does not greatly change the percentage of names that belong to true name pairs, although as expected it does decrease slightly, Appendix Table 7.

| **Context Window Size** | **First 150 names (%)** | **First 200 names (%)** | **First 250 names (%)** | **First 300 names (%)** | **First 350 names (%)** | **First 400 names (%)** | **First 450 names (%)** |
| --- | --- | --- | --- | --- | --- | --- | --- |
| 1 | 6.7 | 6.5 | 6 | 8.7 | 8.3 | 8.3 | 9.3 |
| 3 | 85.3 | 85.0 | 80.0 | 75.7 | 72.0 | 70.0 | 66.7 |
| 5 | 94.7 | 93.0 | 88.0 | 81.0 | 77.7 | 74.8 | 70.7 |
| 7 | 96.7 | 95.0 | 90.0 | 86.3 | 82.0 | 78.5 | 74.2 |
| 9 | 95.3 | 93.5 | 90.8 | 86.3 | 83.1 | 80.0 | 75.8 |
| **Context Window Size** | **First 500 names (%)** | **First 550 names (%)** | **First 600 names (%)** | **First 650 names (%)** | **First 700 names (%)** | **First 750 names (%)** | **First 800 names (%)** |
| 1 | 8.8 | 8.5 | 8.5 | 8.5 | 8.5 | 8.5 | 8.5 |
| 3 | 63.8 | 61.5 | 61.5 | 61.5 | 61.5 | 61.5 | 61.5 |
| 5 | 68.2 | 65.7 | 65.7 | 65.7 | 65.7 | 65.7 | 65.7 |
| 7 | 71.4 | 68.3 | 68.3 | 68.3 | 68.3 | 68.3 | 68.3 |
| 9 | 71.4 | 68.5 | 68.5 | 68.5 | 68.5 | 68.5 | 68.5 |

**Appendix Table 7.** Comparing the effect of choosing a different number of tokens to look at paired tokens, sorted by ascending order, the percentage that are part of existing patient names as a function of context window size, using the cosine distance metric. To determine statistical significance at the patient level, we calculated empirical *p*-values by randomly shuffling all $\binom{n}{2}$ (*n* choose 2) combinations of name tokens 1000 times. All results are significant after correcting for multiple comparisons using Holm-Bonferroni correction.

Note: While iterating through the chosen list names, we disregard names that have been seen before (assuming that they have already been correctly assigned to their first guess). Therefore, when there is no difference between two different settings, it is because newly added pairs (e.g., the 50 new names pairs ranked from 500 to 550) have had one of the pair seen already in the first 500 and are therefore disregarded when gathering statistics.

**Complete Results**

In this section, we present the complete results for all the experiments performed in the paper. We explore varying hyperparameters as well as different measures. Our aim in providing the complete set of results is to demonstrate the robustness and generalizability of our observations across the hyperparameter space as opposed to cherry-picking specific statistics.

**Name Reconstruction Experiment**

| *Context Window Size – Cosine Measure*   \| **Size** \| **Skipgram %-names** \| **CBOW %-names** \| **GLoVe**  **%-names** \| \| --- \| --- \| --- \| --- \| \| 1 \| 8.5 \| 2.8 \| 1.3 ^a^ \| \| 3 \| 61.5 \| 44.2 \| 26.4 \| \| 5 \| 65.6 \| 53.9 \| 46.3 \| \| 7 \| 68.3 \| 55.2 \| 52.8 \| \| 9 \| 68.5 \| 56.7 \| 53.9 \| | *Context Window Size – Cityblock Measure*   \| **Size** \| **Skipgram %-names** \| **CBOW %-names** \| **GLoVe**  **%-names** \| \| --- \| --- \| --- \| --- \| \| 1 \| 8.2 \| 2.8 \| 0.8 ^a^ \| \| 3 \| 50.5 \| 33.6 \| 23.2 \| \| 5 \| 57.3 \| 39.5 \| 39.1 \| \| 7 \| 58.4 \| 40.6 \| 43.6 \| \| 9 \| 59.6 \| 46.5 \| 45.0 \| |
| --- | --- | --- | --- | --- | --- | --- | --- | --- | --- | --- | --- | --- | --- | --- | --- | --- | --- | --- | --- | --- | --- | --- | --- | --- | --- | --- | --- | --- | --- | --- | --- | --- | --- | --- | --- | --- | --- | --- | --- | --- | --- | --- | --- | --- | --- | --- | --- | --- | --- |
| *Embedding Size – Cosine Measure*   \| **Size** \| **Skipgram %-names** \| **CBOW %-names** \| **GLoVe**  **%-names** \| \| --- \| --- \| --- \| --- \| \| 20 \| 56.2 \| 35.7 \| 20.5 \| \| 50 \| 64.3 \| 47.2 \| 40.2 \| \| 100 \| 65.6 \| 53.9 \| 46.3 \| \| 200 \| 66.8 \| 51.6 \| 47.6 \| \| 300 \| 66.8 \| 53.1 \| 47.6 \| | *Embedding Size – Cityblock Measure*   \| **Size** \| **Skipgram %-names** \| **CBOW %-names** \| **GLoVe**  **%-names** \| \| --- \| --- \| --- \| --- \| \| 20 \| 50.7 \| 25.8 \| 18.2 \| \| 50 \| 55.4 \| 36.6 \| 33.4 \| \| 100 \| 57.3 \| 39.5 \| 39.1 \| \| 200 \| 56.7 \| 40.6 \| 41.0 \| \| 300 \| 56.2 \| 42.3 \| 41.4 \| |
| *Learning Rate – Cosine Measure*   \| **Size** \| **Skipgram %-names** \| **CBOW %-names** \| **GLoVe**  **%-names** \| \| --- \| --- \| --- \| --- \| \| 0.0125 \| 65.1 \| 51.0 \| 26.8 \| \| 0.025 \| 65.6 \| 53.9 \| 39.7 \| \| 0.05 \| 65.5 \| 50.5 \| 46.3 \| \| 0.1 \| 66.6 \| 51.8 \| 52.0 \| | *Learning Rate – Cityblock Measure*   \| **Size** \| **Skipgram %-names** \| **CBOW %-names** \| **GLoVe**  **%-names** \| \| --- \| --- \| --- \| --- \| \| 0.0125 \| 57.7 \| 40.6 \| 25.4 \| \| 0.025 \| 57.3 \| 39.5 \| 33.6 \| \| 0.05 \| 56.2 \| 37.4 \| 39.1 \| \| 0.1 \| 56.2 \| 39.5 \| 43.6 \| |
| *Negative Sampling Rate – Cosine Measure*   \| **Size** \| **Skipgram %-names** \| **CBOW %-names** \| \| --- \| --- \| --- \| \| 1 \| 66.4 \| 52.0 \| \| 5 \| 65.6 \| 53.9 \| \| 10 \| 65.8 \| 51.4 \| \| 30 \| 66.0 \| 49.9 \| \| 64 \| 65.3 \| 49.9 \| | *Negative Sampling Rate – Cityblock Measure*   \| **Size** \| **Skipgram %-names** \| **CBOW %-names** \| \| --- \| --- \| --- \| \| 1 \| 55.8 \| 40.8 \| \| 5 \| 57.3 \| 39.5 \| \| 10 \| 55.6 \| 38.5 \| \| 30 \| 54.5 \| 39.1 \| \| 64 \| 56.4 \| 38.1 \| |

**Appendix Table 8**. Of the first 600 paired tokens, sorted by ascending order, the percentage that are part of existing patient names as a function of various hyperparameter setting, using different measures. To determine statistical significance at the patient level, we calculated empirical *p*-values by randomly shuffling all *n* choose 2 combinations of name tokens 1000 times. All results are significant after correcting for multiple comparisons using Holm-Bonferroni correction except for those followed by a superscript ‘a’.

**Name-Diagnostic Code Association Experiment**

| *Context Window Size – Cosine Measure*   \| **Size** \| **Skipgram distance** \| **CBOW distance** \| **GLoVe**  **distance** \| \| --- \| --- \| --- \| --- \| \| 1 \| 0.037 \| 0.027 \| –0.019 \| \| 3 \| 0.020 \| 0.053 \| –0.003 ^a^ \| \| 5 \| 0.009 \| 0.046 \| –0.001 ^a^ \| \| 7 \| 0.000 ^a^ \| 0.025 \| –0.004 ^a^ \| \| 9 \| –0.006 ^a^ \| 0.012 \| –0.001 ^a^ \| | *Context Window Size – Cityblock Measure*   \| **Size** \| **Skipgram distance** \| **CBOW distance** \| **GLoVe**  **distance** \| \| --- \| --- \| --- \| --- \| \| 1 \| 3.91 \| 7.59 \| 4.85 \| \| 3 \| 2.88 \| 28.53 \| 5.69 \| \| 5 \| 2.33 \| 39.55 \| 5.45 \| \| 7 \| 1.84 \| 47.10 \| 5.12 \| \| 9 \| 1.51 \| 51.61 \| 5.54 \| |
| --- | --- | --- | --- | --- | --- | --- | --- | --- | --- | --- | --- | --- | --- | --- | --- | --- | --- | --- | --- | --- | --- | --- | --- | --- | --- | --- | --- | --- | --- | --- | --- | --- | --- | --- | --- | --- | --- | --- | --- | --- | --- | --- | --- | --- | --- | --- | --- | --- | --- |
| *Embedding Size – Cosine Measure*   \| **Size** \| **Skipgram distance** \| **CBOW distance** \| **GLoVe**  **distance** \| \| --- \| --- \| --- \| --- \| \| 20 \| 0.022 \| 0.074 \| –0.005 ^a^ \| \| 50 \| 0.013 \| 0.057 \| 0.004 ^a^ \| \| 100 \| 0.009 \| 0.046 \| –0.001 ^a^ \| \| 200 \| 0.009 \| 0.034 \| 0.003 ^a^ \| \| 300 \| 0.010 \| 0.031 \| 0.002 ^a^ \| | *Embedding Size – Cityblock Measure*   \| **Size** \| **Skipgram distance** \| **CBOW distance** \| **GLoVe**  **distance** \| \| --- \| --- \| --- \| --- \| \| 20 \| 0.82 \| 15.78 \| 1.63 \| \| 50 \| 1.47 \| 26.70 \| 3.83 \| \| 100 \| 2.33 \| 39.55 \| 5.45 \| \| 200 \| 3.68 \| 57.84 \| 7.98 \| \| 300 \| 4.90 \| 71.60 \| 10.25 \| |
| *Learning Rate – Cosine Measure*   \| **Size** \| **Skipgram distance** \| **CBOW distance** \| **GLoVe**  **distance** \| \| --- \| --- \| --- \| --- \| \| 0.0125 \| 0.007 \| 0.039 \| –0.006 ^a^ \| \| 0.025 \| 0.009 \| 0.046 \| –0.006 ^a^ \| \| 0.05 \| 0.009 \| 0.039 \| –0.001 ^a^ \| \| 0.1 \| 0.009 \| 0.043 \| 0.004 ^a^ \| | *Learning Rate – Cityblock Measure*   \| **Size** \| **Skipgram distance** \| **CBOW distance** \| **GLoVe**  **distance** \| \| --- \| --- \| --- \| --- \| \| 0.0125 \| 2.23 \| 39.74 \| 5.80 \| \| 0.025 \| 2.33 \| 39.55 \| 5.63 \| \| 0.05 \| 2.21 \| 39.61 \| 5.45 \| \| 0.1 \| 2.35 \| 40.94 \| 5.12 \| |
| *Negative Sampling Rate – Cosine Measure*   \| **Size** \| **Skipgram distance** \| **CBOW distance** \| \| --- \| --- \| --- \| \| 1 \| 0.008 \| 0.041 \| \| 5 \| 0.009 \| 0.046 \| \| 10 \| 0.010 \| 0.043 \| \| 30 \| 0.009 \| 0.043 \| \| 64 \| 0.006 ^a^ \| 0.038 \| | *Negative Sampling Rate – Cityblock Measure*   \| **Size** \| **Skipgram distance** \| **CBOW distance** \| \| --- \| --- \| --- \| \| 1 \| 2.31 \| 40.24 \| \| 5 \| 2.33 \| 39.55 \| \| 10 \| 2.37 \| 40.11 \| \| 30 \| 2.28 \| 39.62 \| \| 64 \| 2.23 \| 39.62 \| |

**Appendix Table 9**. Difference between the in-group and outgroup as a function of various hyperparameter settings, using different measures. We use the Wilcoxon signed-rank test to compare the pairings of in- and out-groups for each name on the population level. All results are significant after correcting for multiple comparisons using Holm-Bonferroni correction except for those followed by a superscript ‘a’.

| *Context Window Size – Cosine Measure*   \| **Size** \| **Skipgram %patients** \| **CBOW %patients** \| **GLoVe**  **%patients** \| \| --- \| --- \| --- \| --- \| \| 1 \| 6.4 \| 4.2 \| 0 \| \| 3 \| 3.9 \| 5.5 \| 0 \| \| 5 \| 3.3 \| 5.8 \| 0 \| \| 7 \| 3.1 \| 2.8 \| 0 \| \| 9 \| 2.2 \| 2.8 \| 0 \| | *Context Window Size – Cityblock Measure*   \| **Size** \| **Skipgram %patients** \| **CBOW %patients** \| **GLoVe**  **%patients** \| \| --- \| --- \| --- \| --- \| \| 1 \| 7.7 \| 12.1 \| 62.7 \| \| 3 \| 6.4 \| 23.4 \| 62.8 \| \| 5 \| 5.2 \| 23.8 \| 63.2 \| \| 7 \| 2.5 \| 24.0 \| 59.6 \| \| 9 \| 1.9 \| 24.0 \| 70.4 \| |
| --- | --- | --- | --- | --- | --- | --- | --- | --- | --- | --- | --- | --- | --- | --- | --- | --- | --- | --- | --- | --- | --- | --- | --- | --- | --- | --- | --- | --- | --- | --- | --- | --- | --- | --- | --- | --- | --- | --- | --- | --- | --- | --- | --- | --- | --- | --- | --- | --- | --- |
| *Embedding Size – Cosine Measure*   \| **Size** \| **Skipgram %patients** \| **CBOW %patients** \| **GLoVe**  **%patients** \| \| --- \| --- \| --- \| --- \| \| 20 \| 4.4 \| 5.5 \| 0 \| \| 50 \| 3.0 \| 7.0 \| 0 \| \| 100 \| 3.2 \| 5.8 \| 0 \| \| 200 \| 2.4 \| 6.3 \| 0 \| \| 300 \| 2.8 \| 5.6 \| 0 \| | *Embedding Size – Cityblock Measure*   \| **Size** \| **Skipgram %patients** \| **CBOW %patients** \| **GLoVe**  **%patients** \| \| --- \| --- \| --- \| --- \| \| 20 \| 6.3 \| 22.9 \| 68.5 \| \| 50 \| 3.8 \| 22.7 \| 75.2 \| \| 100 \| 5.2 \| 23.8 \| 63.2 \| \| 200 \| 4.1 \| 24.9 \| 58.8 \| \| 300 \| 3.6 \| 24.1 \| 50.8 \| |
| *Learning Rate – Cosine Measure*   \| **Size** \| **Skipgram %patients** \| **CBOW %patients** \| **GLoVe**  **%patients** \| \| --- \| --- \| --- \| --- \| \| 0.0125 \| 3.0 \| 4.6 \| 0 \| \| 0.025 \| 3.3 \| 5.8 \| 0 \| \| 0.05 \| 2.2 \| 3.9 \| 0 \| \| 0.1 \| 3.0 \| 6.1 \| 0 \| | *Learning Rate – Cityblock Measure*   \| **Size** \| **Skipgram %patients** \| **CBOW %patients** \| **GLoVe**  **%patients** \| \| --- \| --- \| --- \| --- \| \| 0.0125 \| 3.9 \| 23.7 \| 62.2 \| \| 0.025 \| 5.2 \| 23.8 \| 65.8 \| \| 0.05 \| 4.6 \| 23.7 \| 63.2 \| \| 0.1 \| 3.9 \| 25.6 \| 65.5 \| |
| *Negative Sampling Rate – Cosine Measure*   \| **Size** \| **Skipgram %patients** \| **CBOW %patients** \| \| --- \| --- \| --- \| \| 1 \| 3.0 \| 5.6 \| \| 5 \| 3.3 \| 5.8 \| \| 10 \| 2.8 \| 5.6 \| \| 30 \| 3.1 \| 5.0 \| \| 64 \| 2.7 \| 4.9 \| | *Negative Sampling Rate – Cityblock Measure*   \| **Size** \| **Skipgram %patients** \| **CBOW %patients** \| \| --- \| --- \| --- \| \| 1 \| 4.9 \| 24.8 \| \| 5 \| 5.2 \| 23.8 \| \| 10 \| 4.2 \| 23.8 \| \| 30 \| 4.6 \| 24.6 \| \| 64 \| 3.9 \| 24.3 \| |

**Appendix Table 10**. The percentage of patients whose diagnoses is identifiable due to a statistically significant difference between in-group and out-group as a function of various hyperparameter settings, using different measures. To determine statistical significant at the patient level, we calculated empirical *p*-values by randomly sampling the in- and out-groups generated using 1000 permutations of the same size from the same dataset.

**Scenario Experiment**

| *Context Window Size – Cosine Measure*   \| **Size** \| **Skipgram A@1, A@5** \| **CBOW A@1, A@5** \| **GLoVe**  **A@1, A@5** \| \| --- \| --- \| --- \| --- \| \| 1 \| 55.8, 56.7 \| 61.8, 61.8 \| 55.4, 56.9 \| \| 3 \| 55.6, 53.1 \| 51.2, 52.6 \| 60.5, 59.5 \| \| 5 \| 57.4, 55.6 \| 53.6, 54.5 \| 59.4, 57.2 \| \| 7 \| 57.4, 53.5 \| 54.6, 53.9 \| 55.9, 54.0 \| \| 9 \| 57.2, 53.2 \| 53.7, 51.2 \| 60.6, 56.7 \| | *Context Window Size – Cityblock Measure*   \| **Size** \| **Skipgram A@1, A@5** \| **CBOW A@1, A@5** \| **GLoVe**  **A@1, A@5** \| \| --- \| --- \| --- \| --- \| \| 1 \| 35.0, 54.1 \| 40.6, 56.8 \| 38.3, 53.7 \| \| 3 \| 43.7, 55.8 \| 31.7, 54.9 \| 40.8, 55.7 \| \| 5 \| 44.1, 55.6 \| 31.9, 58.8 \| 39.5, 54.6 \| \| 7 \| 40.9, 53.3 \| 30.3, 57.7 \| 38.3, 53.8 \| \| 9 \| 43.9, 55.9 \| 32.9, 57.4 \| 45.2, 57.4 \| |
| --- | --- | --- | --- | --- | --- | --- | --- | --- | --- | --- | --- | --- | --- | --- | --- | --- | --- | --- | --- | --- | --- | --- | --- | --- | --- | --- | --- | --- | --- | --- | --- | --- | --- | --- | --- | --- | --- | --- | --- | --- | --- | --- | --- | --- | --- | --- | --- | --- | --- |
| *Embedding Size – Cosine Measure*   \| **Size** \| **Skipgram A@1, A@5** \| **CBOW A@1, A@5** \| **GLoVe**  **A@1, A@5** \| \| --- \| --- \| --- \| --- \| \| 20 \| 58.0, 51.9 \| 48.3, 51.4 \| 60.5, 56.9 \| \| 50 \| 57.1, 52.4 \| 48.7, 55.3 \| 59.1, 55.4 \| \| 100 \| 57.4, 55.6 \| 53.6, 54.5 \| 59.4, 57.2 \| \| 200 \| 56.8, 56.6 \| 56.3, 58.2 \| 60.3, 58.1 \| \| 300 \| 55.0, 55.4 \| 59.2, 57.7 \| 57.9, 57.6 \| | *Embedding Size – Cityblock Measure*   \| **Size** \| **Skipgram A@1, A@5** \| **CBOW A@1, A@5** \| **GLoVe**  **A@1, A@5** \| \| --- \| --- \| --- \| --- \| \| 20 \| 48.8, 55.2 \| 36.3, 56.5 \| 41.3, 56.1 \| \| 50 \| 43.0, 54.2 \| 33.8, 56.6 \| 39.1, 55.4 \| \| 100 \| 44.1, 55.6 \| 31.9, 58.8 \| 39.5, 54.6 \| \| 200 \| 40.3, 56.3 \| 32.0, 57.9 \| 38.7, 56.2 \| \| 300 \| 39.8, 56.4 \| 35.0, 59.8 \| 40.6, 55.4 \| |
| *Learning Rate – Cosine Measure*   \| **Size** \| **Skipgram A@1, A@5** \| **CBOW A@1, A@5** \| **GLoVe**  **A@1, A@5** \| \| --- \| --- \| --- \| --- \| \| 0.0125 \| 59.1, 54.1 \| 54.3, 50.9 \| 55.6, 56.1 \| \| 0.025 \| 57.4, 55.6 \| 53.6, 54.5 \| 58.3, 55.5 \| \| 0.05 \| 54.9, 56.0 \| 51.9, 55.6 \| 59.4, 57.2 \| \| 0.1 \| 54.6, 53.9 \| 56.8, 57.5 \| 59.7, 55.5 \| | *Learning Rate – Cityblock Measure*   \| **Size** \| **Skipgram A@1, A@5** \| **CBOW A@1, A@5** \| **GLoVe**  **A@1, A@5** \| \| --- \| --- \| --- \| --- \| \| 0.0125 \| 41.6, 58.5 \| 30.2, 57.6 \| 38.6, 51.1 \| \| 0.025 \| 44.1, 55.6 \| 31.9, 58.8 \| 38.9, 51.6 \| \| 0.05 \| 45.2, 54.7 \| 30.4, 55.9 \| 39.5, 54.6 \| \| 0.1 \| 45.4, 54.9 \| 32.4, 58.1 \| 40.6, 57.8 \| |
| *Negative Sampling Rate – Cosine Measure*   \| **Size** \| **Skipgram**  **A@1, A@5** \| **CBOW**  **A@1, A@5** \| \| --- \| --- \| --- \| \| 1 \| 58.2, 52.4 \| 55.4, 52.6 \| \| 5 \| 57.4, 55.6 \| 53.6, 54.5 \| \| 10 \| 58.0, 53.2 \| 53.5, 52.4 \| \| 30 \| 58.4, 53.8 \| 53.2, 53.1 \| \| 64 \| 59.2, 54.0 \| 50.1, 55.3 \| | *Negative Sampling Rate – Cityblock Measure*   \| **Size** \| **Skipgram**  **A@1, A@5** \| **CBOW**  **A@1, A@5** \| \| --- \| --- \| --- \| \| 1 \| 36.1, 51.3 \| 32.0, 55.1 \| \| 5 \| 44.1, 55.6 \| 31.9, 58.8 \| \| 10 \| 39.0, 52.9 \| 31.8, 57.4 \| \| 30 \| 43.4, 56.5 \| 33.3, 56.6 \| \| 64 \| 40.4, 54.9 \| 31.8, 59.6 \| |

**Appendix Table 11**. Percentage of times (of 1000 random diagnosis selections) where using a word embedding–based attack beats the majority baseline for A@1 and A@5 for various hyperparameters and distance metrics.

| *Context Window Size – Cosine Measure*   \| **Size** \| **Skipgram A@1, A@5** \| **CBOW A@1, A@5** \| **GLoVe**  **A@1, A@5** \| \| --- \| --- \| --- \| --- \| \| 1 \| 0.02, 0.15 \| 0.04, 0.13 \| 0.02, 0.11 \| \| 3 \| 0.02, 0.12 \| 0.01, 0.14 \| 0.02, 0.13 \| \| 5 \| 0.02, 0.12 \| 0.02, 0.11 \| 0.03, 0.13 \| \| 7 \| 0.01, 0.10 \| 0.02, 0.10 \| 0.02, 0.12 \| \| 9 \| 0.01, 0.11 \| 0.02, 0.11 \| 0.02, 0.12 \| | *Context Window Size – Cityblock Measure*   \| **Size** \| **Skipgram A@1, A@5** \| **CBOW A@1, A@5** \| **GLoVe**  **A@1, A@5** \| \| --- \| --- \| --- \| --- \| \| 1 \| 0.06, 0.12 \| 0.03, 0.11 \| 0.08, 0.15 \| \| 3 \| 0.03, 0.11 \| 0.00, 0.15 \| 0.08, 0.15 \| \| 5 \| 0.05, 0.10 \| 0.02, 0.15 \| 0.08, 0.14 \| \| 7 \| 0.01, 0.13 \| 0.02, 0.15 \| 0.08, 0.15 \| \| 9 \| 0.02, 0.11 \| 0.03, 0.15 \| 0.08, 0.15 \| |
| --- | --- | --- | --- | --- | --- | --- | --- | --- | --- | --- | --- | --- | --- | --- | --- | --- | --- | --- | --- | --- | --- | --- | --- | --- | --- | --- | --- | --- | --- | --- | --- | --- | --- | --- | --- | --- | --- | --- | --- | --- | --- | --- | --- | --- | --- | --- | --- | --- | --- |
| *Embedding Size – Cosine Measure*   \| **Size** \| **Skipgram A@1, A@5** \| **CBOW A@1, A@5** \| **GLoVe**  **A@1, A@5** \| \| --- \| --- \| --- \| --- \| \| 20 \| 0.03, 0.11 \| 0.01, 0.13 \| 0.02, 0.14 \| \| 50 \| 0.02, 0.11 \| 0.01, 0.12 \| 0.02, 0.14 \| \| 100 \| 0.02, 0.12 \| 0.02, 0.11 \| 0.03, 0.13 \| \| 200 \| 0.02, 0.12 \| 0.02, 0.11 \| 0.03, 0.14 \| \| 300 \| 0.02, 0.12 \| 0.03, 0.12 \| 0.03, 0.13 \| | *Embedding Size – Cityblock Measure*   \| **Size** \| **Skipgram A@1, A@5** \| **CBOW A@1, A@5** \| **GLoVe**  **A@1, A@5** \| \| --- \| --- \| --- \| --- \| \| 20 \| 0.01, 0.10 \| 0.01, 0.15 \| 0.08, 0.13 \| \| 50 \| 0.05, 0.12 \| 0.08, 0.15 \| 0.01, 0.15 \| \| 100 \| 0.05, 0.10 \| 0.02, 0.15 \| 0.08, 0.14 \| \| 200 \| 0.05, 0.12 \| 0.03, 0.15 \| 0.08, 0.15 \| \| 300 \| 0.06, 0.12 \| 0.02, 0.17 \| 0.07, 0.15 \| |
| *Learning Rate – Cosine Measure*   \| **Size** \| **Skipgram A@1, A@5** \| **CBOW A@1, A@5** \| **GLoVe**  **A@1, A@5** \| \| --- \| --- \| --- \| --- \| \| 0.0125 \| 0.02, 0.12 \| 0.01, 0.12 \| 0.03, 0.13 \| \| 0.025 \| 0.02, 0.12 \| 0.02, 0.11 \| 0.02, 0.12 \| \| 0.05 \| 0.01, 0.12 \| 0.02, 0.12 \| 0.03, 0.13 \| \| 0.1 \| 0.01, 0.11 \| 0.01, 0.12 \| 0.03, 0.14 \| | *Learning Rate – Cityblock Measure*   \| **Size** \| **Skipgram A@1, A@5** \| **CBOW A@1, A@5** \| **GLoVe**  **A@1, A@5** \| \| --- \| --- \| --- \| --- \| \| 0.0125 \| 0.04, 0.11 \| 0.02, 0.15 \| 0.08, 0.14 \| \| 0.025 \| 0.05, 0.10 \| 0.02, 0.15 \| 0.01, 0.15 \| \| 0.05 \| 0.05, 0.11 \| 0.03, 0.15 \| 0.08, 0.14 \| \| 0.1 \| 0.05, 0.12 \| 0.03, 0.15 \| 0.08, 0.12 \| |
| *Negative Sampling Rate – Cosine Measure*   \| **Size** \| **Skipgram**  **A@1, A@5** \| **CBOW**  **A@1, A@5** \| \| --- \| --- \| --- \| \| 1 \| 0.02, 0.11 \| 0.01, 0.12 \| \| 5 \| 0.02, 0.12 \| 0.02, 0.11 \| \| 10 \| 0.01, 0.11 \| 0.01, 0.11 \| \| 30 \| 0.02, 0.12 \| 0.02, 0.12 \| \| 64 \| 0.01, 0.11 \| 0.00, 0.11 \| | *Negative Sampling Rate – Cityblock Measure*   \| **Size** \| **Skipgram**  **A@1, A@5** \| **CBOW**  **A@1, A@5** \| \| --- \| --- \| --- \| \| 1 \| 0.05, 0.12 \| 0.02, 0.16 \| \| 5 \| 0.05, 0.10 \| 0.02, 0.15 \| \| 10 \| 0.03, 0.11 \| 0.03, 0.15 \| \| 30 \| 0.05, 0.12 \| 0.03, 0.15 \| \| 64 \| 0.02, 0.11 \| 0.03, 0.15 \| |

**Appendix Table** **12**. A@1 and A@5 for the set of diagnosis codes (constipation, diarrhea, vaginitis, sexual dysfunction, urinary infection, herpes genitalis, dementia, anorexia, alcoholism, threatened abortion, and AIDS) for varying hyperparameters and distance measures. The majority baseline is A@1 and A@5 of 0.00 and 0.07.
